# Introducing malaria rapid diagnostic tests in private medicine retail outlets: A systematic literature review

**DOI:** 10.1371/journal.pone.0173093

**Published:** 2017-03-02

**Authors:** Theodoor Visser, Katia Bruxvoort, Kathleen Maloney, Toby Leslie, Lawrence M. Barat, Richard Allan, Evelyn K. Ansah, Jennifer Anyanti, Ian Boulton, Siân E. Clarke, Jessica L. Cohen, Justin M. Cohen, Andrea Cutherell, Caitlin Dolkart, Katie Eves, Günther Fink, Catherine Goodman, Eleanor Hutchinson, Sham Lal, Anthony Mbonye, Obinna Onwujekwe, Nora Petty, Julie Pontarollo, Stephen Poyer, David Schellenberg, Elizabeth Streat, Abigail Ward, Virginia Wiseman, Christopher J. M. Whitty, Shunmay Yeung, Jane Cunningham, Clare I. R. Chandler

**Affiliations:** 1 Clinton Health Access Initiative, Boston, Massachusetts, United States of America; 2 Department of Global Health and Development, London School of Hygiene & Tropical Medicine, London, United Kingdom; 3 Bill and Melinda Gates Foundation, Seattle, Washington, United States of America; 4 US President’s Malaria Initiative, United States Agency for International Development, Washington DC, United States of America; 5 Mentor Initiative, West Sussex, United Kingdom; 6 Research & Development Division, Ghana Health Service, Accra, Ghana; 7 Society for Family Health, Abuja, Nigeria; 8 TropMed Pharma Consulting, Lower Shiplake, Oxfordshire, United Kingdom; 9 Department of Disease Control, London School of Hygiene & Tropical Medicine, London, United Kingdom; 10 Harvard T.H. Chan School of Public Health, Boston, United States of America; 11 Population Services International, Nairobi, Kenya; 12 Ministry of Health, Kampala, Uganda; 13 University of Enugu, Agbani, Enugu State, Nigeria; 14 Malaria Consortium, London, United Kingdom; 15 School of Public Health and Community Medicine, University of New South Wales, UNSW, Sydney, Australia; 16 Department of Clinical Research, London School of Hygiene & Tropical Medicine, London, United Kingdom; 17 Global Malaria Program, World Health Organization, Geneva, Switzerland; Centers for Disease Control and Prevention, UNITED STATES

## Abstract

**Background:**

Many patients with malaria-like symptoms seek treatment in private medicine retail outlets (PMR) that distribute malaria medicines but do not traditionally provide diagnostic services, potentially leading to overtreatment with antimalarial drugs. To achieve universal access to prompt parasite-based diagnosis, many malaria-endemic countries are considering scaling up malaria rapid diagnostic tests (RDTs) in these outlets, an intervention that may require legislative changes and major investments in supporting programs and infrastructures. This review identifies studies that introduced malaria RDTs in PMRs and examines study outcomes and success factors to inform scale up decisions.

**Methods:**

Published and unpublished studies that introduced malaria RDTs in PMRs were systematically identified and reviewed. Literature published before November 2016 was searched in six electronic databases, and unpublished studies were identified through personal contacts and stakeholder meetings. Outcomes were extracted from publications or provided by principal investigators.

**Results:**

Six published and six unpublished studies were found. Most studies took place in sub-Saharan Africa and were small-scale pilots of RDT introduction in drug shops or pharmacies. None of the studies assessed large-scale implementation in PMRs. RDT uptake varied widely from 8%-100%. Provision of artemisinin-based combination therapy (ACT) for patients testing positive ranged from 30%-99%, and was more than 85% in five studies. Of those testing negative, provision of antimalarials varied from 2%-83% and was less than 20% in eight studies. Longer provider training, lower RDT retail prices and frequent supervision appeared to have a positive effect on RDT uptake and provider adherence to test results. Performance of RDTs by PMR vendors was generally good, but disposal of medical waste and referral of patients to public facilities were common challenges.

**Conclusions:**

Expanding services of PMRs to include malaria diagnostic services may hold great promise to improve malaria case management and curb overtreatment with antimalarials. However, doing so will require careful planning, investment and additional research to develop and sustain effective training, supervision, waste-management, referral and surveillance programs beyond the public sector.

## Background

Provision of artemisinin-based combination therapies (ACTs) and other antimalarials to patients without confirmed malaria frequently results in overtreatment, potentially delaying diagnosis and treatment of other causes of illness and reducing availability of ACTs for true malaria cases [1, 2]. Overuse of antimalarials by patients without malaria has been estimated to be half of global demand [3].

Prompted by recommendations from the World Health Organization in 2010 [4], national malaria programs in most endemic countries revised their diagnosis and treatment guidelines to emphasize the use of parasite-based diagnosis of malaria before treatment for all suspected malaria cases [5, 6]. Since then, procurement of malaria rapid diagnostic tests (RDTs) has increased significantly in the public health care sector across much of sub-Saharan Africa [5, 7]. In contrast, availability and use of diagnostic testing in the private medicine retail sector has remained low. Efforts to improve or expand malaria case management in the private sector, as demonstrated in the Affordable Medicines Facility- malaria (AMFm) pilot, focused on treatment delivery, but did not promote the use of diagnostic testing [8]. Evidence shows that RDTs or microscopy are available in less than 20% of pharmacies and drug shops selling antimalarials in six out of eight sub-Saharan African countries surveyed in 2013 or 2014 [9]. Though treatment-seeking practices vary greatly between countries, overall approximately one-third of febrile children obtaining malaria drugs are treated by private providers with limited access to malaria diagnostic services [3].

The private health care sector consists of private not-for-profit and private for-profit health providers, with the latter including private health facilities, diagnostic centers, private medicine retailers and informal practitioners [10]. Private medicine retail outlets (PMRs), a large category of for-profit private health providers in many countries [11], include outlets that specialize in medicines such as pharmacies and drug stores, as well as general stores or itinerant vendors that sell medicines along with other household merchandise [12]. In many countries, PMRs play a dominant role in the distribution and sale of antimalarials [9]. Typically, the outlets that specialize in selling medicines have storefronts, product displays, and a counter. Some may have a small room in the back, separated by a curtain or door, for examinations and treatment. Skills and qualifications vary among staff working in these outlets and include physicians, pharmacists, nurses and drug sellers with little to no formal health training [13]. PMRs are allowed to only carry over the counter drugs and in some cases a limited number of prescription drugs such as antimalarials and certain antibiotics. They are typically not allowed to perform diagnostic services, but government regulations vary amongst countries and are often poorly enforced [14–16].

Given the importance of PMRs as a first source of care and antimalarial treatment, several endemic countries in sub-Saharan Africa and Southeast Asia are considering introducing and scaling up RDTs in these outlets to achieve universal access to prompt parasite based diagnosis prior to treatment [17]. Introducing blood-testing in these outlets is not without controversy, and evidence to guide decisions on how and where to scale up RDTs amongst PMRs is currently lacking [18]. PMRs are often poorly supervised, rarely report into health information systems and are not equipped to manage severe illnesses [12]. Although the procedure to perform RDTs does not require specialized training, operators are required to draw and transfer an exact quantity of blood, apply a specific number of buffer drops, wait the required time before a result can be read (i.e. 15 or 20 minutes) and appropriately dispose of the hazardous infectious waste. Without adequate oversight, public health officials fear that PMRs may misdiagnose patients or not treat patients according to malaria guidelines, providing antimalarials or antibiotics to patients that test negative for malaria [19]. PMRs may also use substandard RDTs, affecting the trust in the result of the test and hence adherence to its results [20]. There is also a concern that improper handling of hazardous waste may lead to the spread of other infectious illnesses [21].

This review identifies and synthesizes available evidence and explores how it can help inform decisions about scaling up RDTs in PMRs.

## Objectives

We undertook a systematic review of published and unpublished intervention studies to evaluate available evidence of the implementation and impact of RDT introduction in PMRs (pharmacies, drug stores, general stores, and/or itinerant vendors that sell medicines along with other household merchandise). The review aimed to:

1. Examine outcomes pertaining to RDT uptake, provider adherence to test results, referral, cost and safety.
2. Review characteristics of each intervention to introduce RDT use (e.g. the length and content of trainings, supervision frequency, referral guidelines, demand generation activities and retail price of RDTs) to explore factors that are associated with RDT uptake and provider adherence to test results.

## Methods

### Registration and eligibility criteria

We followed the Preferred Reporting Items for Systematic Reviews and Meta-Analyses (PRISMA) guidelines (http://www.prisma-statement.org/) and registered with PROSPERO (2013:CRD42013006146). We used the following inclusion criteria:

- Participants: Any PMR providers and their patients
- Interventions: Any introduction of RDTs with or without supporting interventions, where RDTs were performed by PMR staff
- Comparisons: Studies were included whether or not there was a comparison group, and whether or not the comparison group was randomly allocated
- Outcomes: Any measurement of the impact of an intervention to introduce RDTs, such as RDT uptake, provider adherence to test results, recommended retail price or safety protocols

We excluded studies that took place outside of PMRs among other private for-profit, private not-for-profit, and public health care providers (e.g., private health facilities, mission or non-governmental facilities, community health workers, and public health facilities); that reported only on the accuracy of RDTs (such as laboratory-based performance comparisons); where RDTs were not introduced into routine practice (if not performed by outlet staff or used only for reference by a research team); that evaluated the use, presence or proportion of outlets stocking RDTs without implementing any interventions to introduce RDTs; and studies based on hypothetical scenarios or modeling. To increase the evidence base, recent studies yet to be published at the time of the search were also included in the review. Principal investigators from unpublished studies were asked to extract specific testing and treatment outcome data to enable analysis across studies. Principal investigators of published studies were also asked to provide clarifications and data on additional outcomes not reported in the publication.

### Search methods

We performed a systematic literature search of electronic databases on November 16, 2016, including PubMed/Medline, Cochrane Library Online, WHOLIS internet databases, IBSS, Web of Science and Ovid (EMBASE, Global Health, and Journals at Ovid). Studies which were yet to be published were identified at a Roll Back Malaria (RBM) Case Management Working Group, Informal Private Sector Task Force meeting in April 2013 [22] and a consultative working meeting on fever case management in the private health care sector in Africa, organized by ACT Consortium in October 2015 [17].

#### Search terms

Literature searches used synonyms and MESH terms for three concepts (i) ‘malaria’ (ii) ‘rapid diagnostic test’ and (iii) ‘private sector’. No search terms or filters for methods were included. Table 1 provides an overview of the search terms.

**Table 1:** Search terms.

Malaria	Malaria+
Diagnosis	rapid diagnostic test+, RDT+, diagnose, diagnosis, diagnostic+, test+, testing (excludes laboratory trial, travel+)
Private sector	private sector+, commerce+, commercial sector, retail sector, private provider, private providers, drug seller, drug sellers, private outlet, private outlets, drug vendor, drug vendors, drug shop, drug shops, retailer, retailers, medicine shop, medicine shops, drug store, drug stores, pharmacy, pharmacies, informal provider, informal providers, patent medicine vendor, retail+, private, drug retailer+, sale+, over-the-counter, unregulated, shop+, profit, informal, chemists, private laboratories

#### Study selection

For published studies the resulting titles and abstracts were reviewed independently by two authors (TV and KB) to select papers or reports to read in full text. Discrepancies were resolved by a third author (KM). Papers that were clearly irrelevant were excluded after reading title and abstract. The remaining papers were read in full and excluded if they did not match the inclusion criteria after agreement between TV, KB and KM. Remaining papers were included in the systematic review.

For inclusion of unpublished studies, investigators were contacted initially to ascertain whether studies met the eligibility criteria, whether data would be available and/or computed within a given time frame and to reach agreement with investigators to include their unpublished findings in the review. Studies that met each of these criteria were subsequently included in the review and investigators asked to contribute results from their studies.

### Data outcomes and extraction

Data extraction tables were used to collate information from both published and unpublished studies. The following diagnosis and treatment outcomes were compared across studies:

1. Uptake: the proportion of patients seeking treatment for fever or suspected malaria who were tested with an RDT
2. RDT positivity: the proportion of patients receiving a positive RDT result
3. ACT provision: the proportion of patients seeking treatment for fever or suspected malaria who were sold ACTs, regardless of whether or not they were tested
4. Adherence to negative or positive test results: the proportion of patients that were sold ACTs in the presence of a positive RDT result or the proportion of patients that that were *not* sold ACTs or other antimalarials in the presence of a negative RDT result
5. Antibiotic provision: the proportion of patients who were sold antibiotics in the presence of a positive RDT result; or the proportion of patients who were sold antibiotics in the presence of a negative RDT result
6. Referrals: the proportion of patients referred to a public facility by the provider for further care
7. Accuracy and safety: the proportion of PMR providers who accurately performed the RDT, read the result, and adequately disposed of the infectious hazardous waste
8. Median retail price of a RDT

We reported outcomes as proportions with comparable denominators where possible. In studies that provided cluster and individual level outcomes, we chose to use individual outcomes to enable comparison across studies. Where the same outcome was reported by more than one method of data collection, we chose the most complete data set, or presented neither if results for an outcome substantially differed between methods. To explore factors that appear to have supported RDT uptake and provider adherence to test results, outcomes across study arms were reviewed in terms of the characteristics of each intervention (length and content of trainings, supervision frequency, demand generation activities, recommended RDT retail price and referral policy). We did not make statistical comparisons between studies because of the different methodologies and outcomes used.

## Results

### Study selection

**Fig 1:**
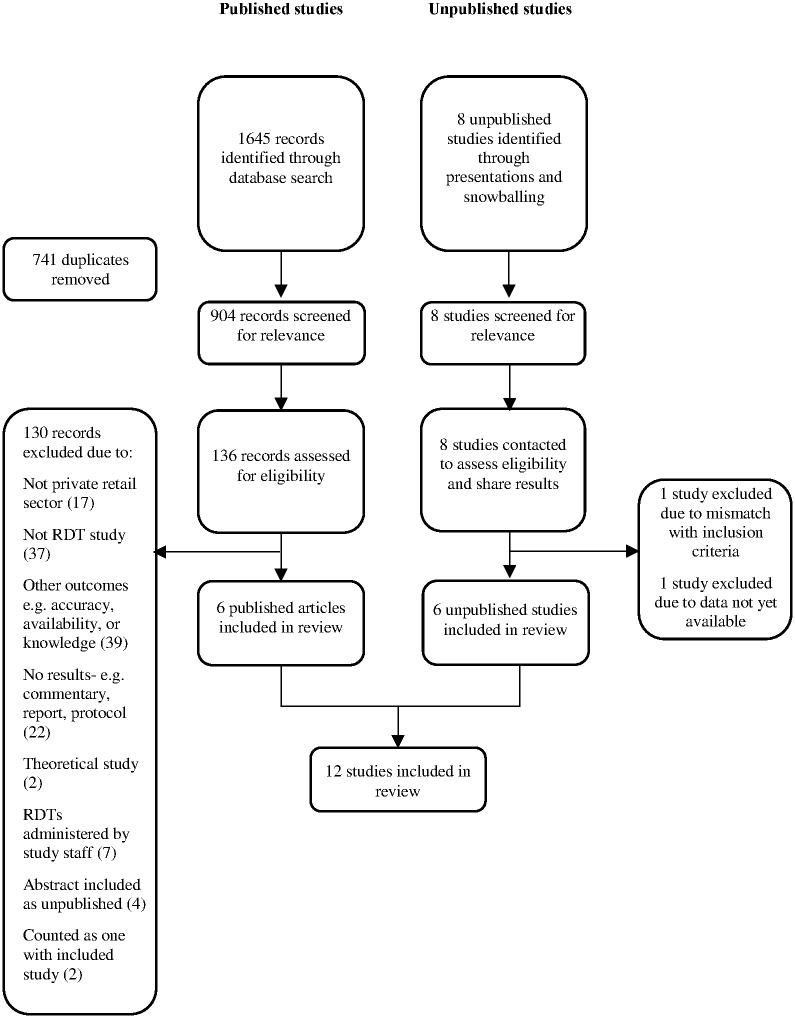
Search strategy.

A total of 1645 titles from published studies were identified through the search strategy (Fig 1). After removing duplicates, 904 titles and abstracts were screened and 136 publications were reviewed in detail. Of these, two studies focused on Cambodia [23, 24], where RDTs had been scaled-up for over a decade. However, these studies did not directly evaluate the impact of implementation of RDTs on any outcomes comparable with other studies. Two other studies (Cohen *et al*. 2012 and Awor *et al*. 2014) published initial data followed by more recent publications with additional data from the same studies (Cohen *et al*. 2015 and Awor *et al*. 2015). Each of these pairs are presented together and counted as one study. In addition, eight unpublished studies were identified. Two of these, a study in Madagascar and a study in Angola, were excluded, as data were not available at the time of this review. In all, six published [25–32] and six unpublished studies (please refer to the supporting information file S2 File) were included in the review, for a total of 12 studies.

### Study design and characteristics

Table 2 provides characteristics of the included studies. Most were trials of pilot interventions to introduce RDTs in PMRs specialized in selling drugs (e.g., drug shops or pharmacies) in sub-Saharan Africa, with one trial in Myanmar (Aung *et al*. 2015). Four studies had a control group without RDTs (Ansah *et al*. 2015; Awor *et al*. 2013; Maloney *et al*. under review; Mbonye *et al*. 2015) and three studies had multiple RDT intervention arms (Aung *et al*. 2015; Maloney *et al*. under review; Onwujekwe *et al*. 2015). The studies took place in areas of medium to high malaria transmission [33] and in rural, peri-urban, and urban settings. Outcomes were assessed using various data collection methods: provider records, exit interviews, mystery shoppers, direct observation, supervision visits, and household surveys (Table 3). Regulations in all study countries except Myanmar did not permit RDTs to be performed by providers in PMRs; studies were granted waivers or special permission from governments. The length of the studies ranged from six months (Onwujekwe *et al*. 2015) to 27 months (Allan *et al*. unpublished data). The number of outlets where RDTs were introduced varied from 29 to over 600 outlets in the intervention arm in Uganda and Myanmar, respectively (Mbonye *et al*. 2015, Aung *et al*. 2015).

**Table 2:** Study characteristics.

Study Design				Study characteristics			Supporting interventions					
#	First author, Country, Year published	Year performed, Length of study	Type of study	Endemicity level*(Low = <5%, Medium =, 5<x<40% High = >40%)	Urban/ Rural	Type and number of outlets included in intervention arm(s)	Sharp box and/or gloves provided, free of cost?	Length and content of provider training	Guidelines for patients that test negative	Supervision frequency and method	Demand generation activities	Were RDTs subsidized? What was Recommended Retail Price RDT
1	Allan, Liberia, Unpublished data	2012–2014, 27 months	Implementation trial	Medium	Urban	Registered medicine store (89), Pharmacy (18)	Yes, both	**3 days**, Content based on MoH approved 'Training Manual for Malaria Case Management in the Private Sector: Pharmacies and Medicine Stores'. Sessions included RDT practice and role play	Refer RDT negative patients to public facility	**Monthly**, direct observation with a checklist by study team At the beginning of every month, a second visit was performed by the supervisors to collect ledger data with tests performed and treatment sold.	40 community volunteer health promotors promoted malaria testing and treatment at the PMRs and through household visits. Mass awareness activities (2 hours of dance, music, theater and games)	Yes, US$0.26
2	Ansah, Ghana,2015	2011–2013, 17 months	Randomized Control Trial	High	Rural / peri-urban	Chemical shop (27)	Yes, only sharp box	**4 days**, Covered malaria symptoms, antimalarial treatment policy, taking blood smears, safety, and RDT use	Refer RDT negative patients to public facility	**Weekly** (for the first month, then another visit midway through trial). Direct observation with a checklist by study team	Community sensitization meetings using short films showing patients going into a chemical shop and complaining of fever and provider performing the RDT.	Yes, offered to patients for free
3	Aung, Myanmar, 2015	2013, 18 months	Randomized Control Trial	High	Rural	General retail stores (398); itinerant drug vendors (177); medical drug representatives (56)		**0.5 days**, Covered RDT procedure (demonstration with performing RDTs), safety, result reading and interpretation, follow up treatment	Refer RDT negative patients to public facility	**Monthly or every 2 weeks** depending on intervention arm. Direct observation by medical detailers using check list	No demand generation activities	Yes, US$0.38
4	Awor, Uganda, 2014, 2015	2011–2012, 9 months	Non-randomized controlled trial	High	Rural	Drug shops (40)	No	**5 days,** Covered integrated Community Case Management and use of RDTs	iCCM	**Daily**, direct observation and feedback by study nurse present during opening hours	Communication campaign through shop branding, provision of information to caretakers and community	Yes, offered to patients for free
5	Cohen, Uganda, 2012, 2015	2011–2012, 12 months	Implementation trial	High	Peri-urban, rural	Drug stores (92)	Yes, both	**2 days**, Covered malaria symptoms, first-line antimalarial treatment, and RDTs (but no specific treatment algorithms)	No guidelines were provided as to how to manage RDT negative patients provided by research team	**Monthly**, direct observation by study team using checklist	Behavioral Change Campaign (BCC) campaign rolled out by a third party at the end of the study period	Yes, but no RRP was provided
6	Maloney, Tanzania, Under review	2013–2014, 12 months	Non-randomized controlled trial	Medium	Rural	Accredited Drug Dispensing Outlets (310)	Yes, both. Note, gloves were added to RDT box by RDT manufac-turer	**2 days,** Trainings covered signs and symptoms of uncomplicated and severe malaria, stocking, use, and disposal of RDTs, and appropriate case management based on RDT results.	Refer customers with signs or symptoms of severe illness and suspected malaria patients who tested negative or whose illness did not improve within 48 hours	**Quarterly**, direct observation by research team using check list	Study coincided with national radio and television campaign promoting RDT use prior to providing treatment	Yes, in one arm of the study, RRP US$0.31, In the other arm, RDTs were not subsidized, RRP of US$0.67
7	Mbonye, Uganda, 2015	2010–2011, 14 months	Randomized Control Trial	High	Peri-urban and rural	Class C drug shops (29)	Yes, both	**4 days**, Covered malaria symptoms, treatment with ACT and rectal artesunate, management of stocks and records, and RDTs, referral policy	Refer RDT negative patients to public facility	**Weekly** (for first 2 months), direct observation by research team using check list. After 2 months scaled-down to support of providers experiencing difficulties	Community sensitization on diagnostic testing and roadside signage for drug shops with RDTs	Yes, fixed price of US$0.2
8	Onwujekwe, Nigeria, 2015	2012, 6 months	Randomized Control Trial	High	Urban, peri-urban, and rural	Patent Medicine Vendors (44) and pharmacy shops (71)	Yes, both	**1 day** (In basic RDT intervention arm) **2 day** (In provider and school-based RDT intervention arms), Covered safe use of RDTs (all arms), plus additional interactive training on malaria diagnosis and treatment for provider and school-based intervention arms	Refer RDT negative patients to public facility	**Monthly or quarterly** (depending on intervention arm), Quarterly visits in the basic RDT intervention arm; monthly visits in the provider and school-based RDT intervention arms by study team using checklists	In school-based intervention arm, teachers were trained on malaria diagnosis and treatment and expected to train students, provide peer educators and conduct community awareness activities	Yes, US$0.6
9	Poyer, Kenya, Unpublished data	2014–2016, 24 months	Implementation trial	Medium	Urban	Pharmacies (44)	Yes, both	**3 days**, Covered malaria epidemiology, testing methods with emphasis on RDT treatment guidelines, commodity management and documentation and reporting.	Abbreviated version of iCCM	**Quarterly** (based on performance), direct observation using a checklist that assigned score to each outlet (based on assessment of danger signs, RDT procedure, provision of treatment and counseling of patient) used to determine the timing of the next visit.	Small group communication sessions and household visits. National media campaign promoting the use of RDTs	No, US$1.17
10	Streat, Nigeria, Unpublished data	2015–2016, 19 months	Implementation trial	Low/ Medium	Urban and rural	Clinics and pharmacies and Proprietary Patent Medicine Vendors (PPMVs) (350 at start of study, 898 at the end of the study),	Yes, only sharp box	**2 days**, e learning covering 'Why testing', Febrile case management, RDT procedure and interpretation, Inter Personal Communications (IPC) skills, waste management, storage of RDTs and training of supervisors, inventory skills, reporting and documentation	Refer to public facility when no capacity to treat	**Quarterly**, direct observation by representatives of professional associations. In addition, a mobile app was used to collect data on stocks and sales of RDTs	Mass media: TV and radio campaigns	Yes, US$1.25
11	Streat, Uganda, Unpublished data	2014–2016, 21 months	Implementation trial	Low/ Medium	Urban and rural	Clinics and pharmacies (and drug shops (150 at start of study, 1502 at the end of the study)	Yes, only sharp box	**2 days**, e learning covering 'Why testing', Febrile case management, RDT procedure and interpretation, IPC skills, waste management, storage of RDTs and training of supervisors, inventory skills, reporting and documentation	Refer to public facility when no capacity to treat	**Quarterly**, direct observation by representatives of professional associations. In addition, a mobile app was used to collect data on individual case data and stocks and sales of RDTs	Roadshows, promotions, mass media TV billboards radio	Yes, US$1
12	Streat, Zambia, Unpublished data	2010–2011, 12	Implementation trial	Medium	Urban and rural	Drug shops (63), pharmacies and grocery stores (40)	Yes, both	**3 days**, Covered malaria epidemiology, RDT procedure and interpretation treatment of positive cases and referral guidelines, commodity management and documentation and reporting, business skills	Refer RDT negative patients to public facility	**Monthly**, direct observation by field supervisors for competency and monthly for stock management	Radio messages with shop name mentioned, launch event (including media coverage), Community meetings	Yes, fixed price of US$0.22

* Endemicity is measured as the percent of people in a community who are infected with malaria parasites at a given point in time. The classification is based on Malaria ATLAS project (http://www.map.ox.ac.uk/explore/about-malaria/malaria-endemicity/)

**Table 3:** Data collection methods.

Study		Outcomes							
First author	Country	Uptake	Positivity	ACT consumption	Adherence	Antibiotic usage	Referrals	Safety & Accuracy	Retail Price
		(% of treatment seeking patients receiving RDT)	(% of patients receiving an RDT who tested positive)	(% of patients presenting with fever who got an ACT)	(% of those testing negative/positive/not tested receiving ACT or AM)	(% of febrile patients testing positive/negative taking antibiotic)	(% of patients referred elsewhere by the provider for further care)	(% of providers who could accurately perform an RDT/ interpret results/dispose of waste)	(Median retail price in US$)
Allan	Liberia	Mystery shopper	Mystery shopper	Mystery shopper	Exit interviews	Mystery shopper	Mystery shopper	Mystery shopper	Mystery shopper
Ansah	Ghana	Provider records	Provider records	Provider records	Provider records	Provider records	Provider records	Direct observations	NA
Aung	Myanmar	Mystery shopper	Mystery shopper	Mystery shopper	NA	NA	NA	Mystery shopper	Mystery shopper
Awor	Uganda	Exit interviews	Exit interviews	Exit interviews	Direct observations	NA	NA	NA	NA
Cohen	Uganda	Provider records	Provider records	Monthly household surveys	Monthly household surveys	Monthly household surveys	NA	Supervision visits	Supervision visits
Maloney	Tanzania	Exit interviews	Exit interviews	Exit interviews	Exit interviews	Exit interviews	NA	Supervision visits	Supervision visits
Mbonye	Uganda	Provider records	Provider records	Provider records	Provider records	Household follow up surveys	Provider records	Supervision visits	Household follow up surveys
Onwujekwe	Nigeria	Exit interviews	Exit interviews	Exit interviews and provider records	Exit interviews and provider records	Exit interviews and provider records	Exit interviews	NA	Exit interviews
Poyer	Kenya	Exit interviews	Exit interviews	Exit interviews	Exit interviews	Exit interviews	Exit interviews	NA	Exit interviews
Streat	Nigeria	Provider records and exit interviews	Provider records	NA	Provider records	NA	NA	Supervision visits	Exit interviews
Streat	Uganda	Exit interviews	Exit interviews	Exit interviews	Exit interviews	NA	NA	Supervision visits	Exit interviews
Streat	Zambia	Exit interviews	Exit interviews	NA	Mystery shopper	Mystery shopper	NA	Mystery shopper	Exit interviews

RDTs were either free to patients (Ansah *et al*. 2015; Awor *et al*. 2014, 2015) or heavily subsidized by implementers. Subsidies ranged from US$0.26 to US$0.8 per RDT (Mbonye *et al*. 2015; Aung *et al*. 2015; Streat *et al*. Zambia unpublished data; Streat *et al*. Ug unpublished data; Streat *et al*. Nigeria unpublished data; Allan *et al*. unpublished data; Onwujekwe *et al*. 2015; Cohen *et al*. 2012, 2015), except in a study in Kenya (Poyer *et al*. unpublished results) and one arm of a study in Tanzania (Maloney *et al*. under review), where RDTs were not subsidized. Gloves and infectious hazardous waste disposal units (i.e. a sharp box) were provided free of cost in most studies. RDTs were distributed either directly to a participating provider from a research warehouse or through a pre-selected wholesaler, importer or government entity. The length of the training varied from half a day to five days and often combined lectures and practice in performing the RDTs. Training content typically covered the symptoms of malaria and the recommended policies on antimalarial treatment and safety. In most intervention arms, training emphasized adherence to test results, including guidance on referral to nearby public health facilities for patients with signs of severe disease. Most studies also recommended referral when patients tested negative. Exceptions included a study in Uganda, where providers were trained on ACTs as first-line malaria treatment and how to perform RDTs but were not given specific algorithms on when to use RDTs or how to manage positive and negative results (Cohen *et al*. 2012, 2015). In another study in Uganda, the RDT introduction was part of a five day integrated community case management (iCCM) training that included treatment of malaria, pneumonia, and diarrhoea (Awor *et al*. 2014, 2015). In one arm of a study in Nigeria, training focused only on how to perform RDTs (Onwujekwe *et al*. 2015). The frequency of supportive supervision also differed, but in most studies research staff visited participating facilities monthly or quarterly to evaluate stock management, waste management practices and how RDTs were performed, stored, and priced (through direct observation using a checklist). In a study in Kenya, outlet supervision visits were conducted based on febrile client volume and quality of care, prioritizing high volume and under-performing outlets (Poyer *et al*. unpublished data). In studies in Nigeria and Uganda, professional associations and medical retailers were contracted to perform the supervision (Streat *et al*. Nigeria unpublished data; Streat *et al*. Uganda unpublished data). Activities to raise awareness of RDTs included organizing community meetings and door to door visits using volunteer health promotors (Allan *et al*. unpublished data, Mbonye *et al*. 2015), community films (Ansah *et al*. 2015) conducting small group communication sessions and household visits (Poyer *et al*. unpublished data), school-based activities (Onwujekwe *et al*. 2015) and national or regional media campaigns promoting the use of RDTs (Cohen *et al*. 2012, 2015; Maloney *et al*. under review; Poyer *et al*. unpublished data; Streat *et al*. Nigeria unpublished data; Streat *et al*. Uganda unpublished data).

### Testing and treatment outcomes

Table 4 provides the diagnosis and treatment outcomes included in the review. Table 5 provides a summary of diagnosis and treatment outcomes by study arm alongside a summary of the supporting interventions implemented, ordered by RDT uptake (high to low).

**Table 4:** Diagnosis and treatment outcomes.

Study			Outcomes										
			Percent (Numerator / Denominator)										
Author	Country	Description of intervention arm (s)	RDT uptake	RDT positivity	ACTs dispensed	Adherence			Antibiotics provided		Referrals	Safety & Accuracy	Cost
			% of treatment seeking patients receiving RDT	% of patients receiving an RDT who tested positive	% of treatment seeking patients who received an ACT	% of patients with a negative RDT result not receiving ACT or AM	% of patients with a positive RDT result receiving ACT	% of patients not tested receiving ACT or other AM	% of patients with a negative RDT result receiving antibiotic	% of patients with a positive RDT result receiving antibiotic	% of patients referred elsewhere by the provider for further care	% of providers who could accurately perform an RDT, read its result and dispose of waste (at the time of performing the RDT)	(Median retail price to patient in US$)
Allan	Liberia	Trained provider and subsidized RDTs	41 (38/92)	36 (29695/81530)	36 (33/92) ^a^	79 (30/38) ^a^	79 (15/19)^a^	74 (40/54) ^a^	11 (4/38) ^a^	NA	10 (9/92)	39 (15/38)	0.32
Ansah	Ghana	Intervention arm: Trained providers and subsidized RDTs	100 (2719/2719)	49.7 (1351/2719)	47 (1247/2641)	97 (1330/1368)	99.5 (1344/1351)	NA	0.65 (8/1368)	0 (0/1351)	40 (1095/2719)	87.2–100 (116/133)^e^	Free
		Control arm: Trained providers but no RDTs	NA	NA	83 (1632/1962)	NA	NA	NA	NA	NA	0 1/2029	NA	NA
Aung	Myanmar	Arm 1: RDT subsidy and resupply in exchange for used RDTs plus monthly check-in visit	51 (32/63) ^a^	NA	NA	80 (28/35)^b^	NA	NA	NA	NA	NA	94^d^ (110/116)	NA
		Arm 2: Price subsidy plus free RDT kit for every five purchased	64 (35/55) ^a^	NA	NA	83 (30/36) ^b^	NA	NA	NA	NA	NA	NA	NA
		Arm 3: Price subsidy, bimonthly support and education visits	59(31/53) ^a^	NA	NA	87(39/45) ^b^	NA	NA	NA	NA	NA	NA	NA
Awor	Uganda	Intervention arm: Trained providers in iCCM with malaria RDTs, with provision of drugs	87.7 (427/497)	75	81 (393/487)	91 (10/11)	100 (33/33)	NA	NA	NA	NA	NA	NA
		Control arm: No iCCM training and provision of ACTs only	NA	NA	41 (113/275)	NA	NA	NA	NA	NA	NA	NA	NA
Cohen	Uganda	Trained provider and subsidized RDTs	17 (478/2235)	89 (421/475)	29 (840/2868)	59 (32/54)	30 (128/421)	60 (1414/2362)	31 (17/54)	31 (129/441)	NA	99 (273/275)	0.4
Maloney	Tanzania	Intervention arm: Trained providers and subsidized RDTs	66 (143/217)	41	32 (60/185)	91 (69/76)	84 (38/45)	57 (36/63)	11 (8/76)	16 (7/45)	NA	90 (165/184)^c^	0.31
		Intervention arm: Trained providers and unsubsidized RDTs	65 (160/247)	41	32 (68/211)	95 (76/80)	67 (35/52)	82 (64/78)	10 (8/80)	6 (3/52)	NA	NA	0.67
		Control arm: No training or RDTs	N/A	NA	43 (83/192)	NA	100 (7/7)	72 (133/184)	NA	57 (4/7)	NA	NA	NA
Mbonye	Uganda	Intervention arm: Trained providers and subsidized RDTs	97.8 (8480/8672)	58.5	60.8 (4907/8073)	98.5 (3117/3166)	99.0 (4858/4907)	NA	45.1 (51/113)	23.6 (30/127)	11.2 (839/7522)	95 (6931/7270) ^d^	0.2
		Control arm: Trained providers but no RDTs	NA	NA	99.7 (6781/6797)	NA	NA	99.7 (6781/6797)	NA	NA	3.3 (189/5797)	NA	NA
Onwujekwe	Nigeria	Intervention arm: Demonstration on how to use RDTs and subsidized RDTs	25 (335/1352)	75	48 (642/1352)	45 (27/60)	76 (193/254)	89 (805/907)	12 (7/58)	17 (41/249)	0.8 (11/1316)	NA	0.9
		Intervention arm: Trained providers and subsidized RDTs	12 (185/1510)	33	49 (733/1510)	43 (50/117)	80 (55/69)	86 (1121/1307)	17 (20/116)	15 (10/68)	0.8 (12/1502)	NA	0.9
		Intervention arm: Trained providers, subsidized RDTs, plus school-based intervention	8 (109/1292)	42	56 (722/1292)	17 (8/48)	71 (43/61)	90 (1045/1159)	17 (8/48)	11 (7/61)	0.8 (10/1276)	NA	1.2
Poyer	Kenya	Intervention arm: RDTs in pharmacies	34.2 (41/121)	47.7 (20/41)	46.8 (56/120)	84.8 (17/20)	84.4 (17/20)	50.0 (34/69)	36.4 (7/20)	18.9 (3/20)	7.9 (9/120)	NA	1.14
Streat	Nigeria	Intervention arm: Trained providers and subsidized RDTs	NA	33 (4812/14619)	NA	87 (7881/9028)	88 (4238/481	NA	NA	NA	NA	72 (192/268)	1.5
Streat	Uganda	Intervention arm: Trained providers and subsidized RDTs	48 (802/1671)	70 (154/221)	NA	84 (799/952)	83 (261/315)	NA	NA	NA	NA	75 (351/469)	1
Streat	Zambia	Trained providers and subsidized RDTs	72 (130/178)	73 (101/138)	27 (69/256)	77 (34/44) ^a^	87 (88/101) ^a^	62 (142/228) ^a^	3 (3/101)	2 (2/94) ^a^	NA	90 (29/32)	0.2

^a^) Based on mystery shopper survey which did not prompt for RDT, ACT or antibiotic

^b^) Assumes all mystery shoppers were tested RDT negative

^c^) Reported proportion of health providers reading test accurately separately. Results show similar high scores: 98% (180/184)

^d^) Waste disposal procedure was not included in the assessment

^e^) 87.2–100 indicates the range of outcomes for each of the indicators. Out of 133 observations, 116 represent the number of chemical sellers who immediately discarded the sharps into the sharps bin

**Table 5:** Summary of diagnosis and treatment outcomes^a^.

Study			Outcomes				Supporting interventions				
First author	Country	Intervention arm description	Uptake	Adherence to negative result	Adherence to positive result	Safety & Accuracy	Length of provider training	Supervision frequency	Median observed retail price to patient	Guidance to providers on treatment after RDT negative test result	Promo-tional activities
			(% of treatment seeking patients receiving RDT)	(% of those testing negative not receiving ACT or AM)	(% of those testing positive receiving ACT)	Providers who could accurately perform an RDT, read its result and dispose of waste (at the time of performing the RDT)	(in days)				
Ansah	Ghana	Trained providers and subsidized RDTs	100%	97%	99%	NA	4 days	Weekly (for 1 month)	US$0	Yes	Yes
Mbonye	Uganda	Trained providers and subsidized RDTs	98%	98%	99%	95%	4 days	Weekly (for 2 months)	US$0.2	Yes	Yes
Awor	Uganda	Trained providers in iCCM with malaria RDTs and drugs	88%	91%	100%	NA	5 days	Daily	US$0	Yes	Yes
Streat	Zambia	Trained providers and subsidized RDTs	72%	77%	87%	90%	3 days	Monthly	US$0.2	Yes	Yes
Aung	Myanmar	Arm 2: Price subsidy plus free RDT kit for every five purchased	66%	83%	NA	97%	0.5 days	Monthly	US$0.38	Yes	No
Maloney	Tanzania	Arm 1: Trained providers and subsidized RDTs	66%	91%	84%	90%	2 days	Quarterly	US$0.31	Yes	Yes
Maloney	Tanzania	Arm 2: Trained providers and unsubsidized RDTs	64%	95%	67%	87%	2 days	Quarterly	US$0.67	Yes	Yes
Aung	Myanmar	Arm 3: Price subsidy, bimonthly support and education visits	59%	87%	NA	94%	0.5 days	Every 2 weeks	US$0.38	Yes	Yes
Aung	Myanmar	Arm 1: RDT subsidy and resupply in exchange for used RDTs plus monthly check-in visit	51%	80%	NA	86%	0.5 days	Monthly	US$0.38	Yes	No
Streat	Uganda	Trained providers and subsidized RDTs	48%	84%	83%	72%	2 days	Quarterly	US$1.5	Yes	Yes
Allan	Liberia	Trained provider and subsidized RDTs	41%	79%	79%	16%	3 days	Monthly	US$0.32	Yes	Yes
Poyer	Kenya	Trained providers and subsidized RDTs	34%	85%	85%	NA	3 days	Quarterly (based on performance)	US$1.17	Yes	Yes
Onwujek-we	Nigeria	Arm 1: Demonstration on how to use RDTs and subsidized RDTs	25%	45%	76%	NA	1 days	Quarterly	US$0.9	Yes	No
Cohen	Uganda	Trained provider and subsidized RDTs	17%	59%	30%	99%	2 days	Monthly	US$0.4	No	Yes
Onwujek-we	Nigeria	Arm 2: Trained providers and subsidized RDTs	12%	53%	80%	NA	2 days	Monthly	US$0.9	Yes	Yes
Onwuje-kwe	Nigeria	Arm 3: Trained providers and subsidized RDTs & plus school-based intervention	8%	17%	71%	NA	2 days	Monthly	US$1.2	Yes	No
Streat	Nigeria	Trained providers and subsidized RDTs	NA	81%	88%	75%	2 days	Quarterly	US$1	Yes	Yes

^a^) Ordered for ‘Uptake’ from high to low. Color coding of the different outcomes and interventions is based on the relative magnitude of the outcome (i.e., higher uptake is green, lower uptake is red) and the relative intensity of the intervention (i.e., shorter trainings are red, longer trainings are green).

#### Uptake of RDTs

All studies reported on uptake (the proportion of eligible patients for whom an RDT was undertaken), which ranged from 8% (96/1279, exit interviews) in the provider and school-based intervention arm of a study in Nigeria (Onwujekwe *et al*. 2015) to 100% (2719/2719, provider records) in a study in Ghana (Ansah *et al*. 2015). Five studies reported uptake below 50% (Cohen *et al*. 2013, 2015; Streat *et al*. Uganda unpublished data; Allan *et al*. unpublished data; Onwujekwe *et al*. 2015; Poyer *et al*. unpublished), three studies reported uptake between 50% and 80% (Aung *et al*. 2015; Maloney *et al*. under review; Streat *et al*. Zambia unpublished data) and three studies reported uptake above 80% (Mbonye *et al*. 2015; Ansah *et al*. 2015; Awor *et al*. 2014, 2015).

#### ACT provision and RDT positivity

Eight studies reported on ACT provision among all patients seeking treatment for fever or suspected malaria. ACT provision ranged from 30% to 60% in six studies (Allan *et al*. unpublished data; Ansah *et al*. 2015; Maloney *et al*. under review; Mbonye *et al*. 2015; Onwujekwe *et al*. 2015; Poyer *et al*. unpublished data). Both the highest ACT provision (81%, 393/487, exit interviews) and the lowest (29%, 840/2868, household surveys) were reported in studies in Uganda (Awor *et al*. 2014, 2015 and Cohen *et al*. 2012, 2015, respectively).

ACT provision was compared between intervention and control (no RDT intervention) arms in four studies: in Uganda (Mbonye *et al*. 2015; Awor *et al*. 2014, 2015), Ghana (Ansah *et al*. 2015), and Tanzania (Maloney *et al*. under review). In three of these studies, 10 to 40 percentage points fewer patients in the RDT intervention arms compared to the control arms obtained ACTs (Mbonye *et al*. 2015, Ansah *et al*. 2015; Maloney *et al*. under review). RDT positivity in these studies was approximately 50% to 60%. In Awor *et al*. (2014, 2015), 20 percentage points more patients in the intervention arm compared to the control arm obtained ACTs. In this study, RDT positivity and ACT provision were both high (75% and 81%, respectively). Four other studies also reported similarly high RDT positivity (Streat *et al*. Uganda, unpublished data; Streat *et al*. Zambia, unpublished data; Onwujekwe *et al*. 2015; Cohen *et al*. 2012, 2015), while RDT positivity in the remaining studies ranged from 33% to 50% (Ansah *et al*. 2015; Mbonye *et al*. 2015; Onwujekwe *et al*. 2015; Allan *et al*. unpublished data).

#### Adherence to RDT results

All studies reported adherence to RDT results. Overall, antimalarials were less commonly provided to RDT-negative patients than to RDT-positive and untested patients. In eight studies, the percentage of RDT-negative patients who received an unnecessary antimalarial was at or below 20% (Ansah *et al*. 2015; Awor *et al*. 2014, 2015; Aung *et al*. 2015; Maloney *et al*. under review; Mbonye *et al*. 2015; Poyer *et al*. unpublished data; Streat *et al*. Nigeria unpublished data; Streat *et al*. Uganda unpublished data). However, high adherence was not universal. A study in Uganda (Cohen *et al*. 2013, 2015) found relatively low adherence, with 41% of patients testing negative receiving an antimalarial (22/54 household members reporting getting an RDT at a drug shop). In addition, all three intervention arms in a study in Nigeria (Onwujekwe *et al*. 2015) found very low adherence, with over 50% of those testing negative receiving an antimalarial.

The proportions of RDT-positive patients receiving ACTs exceeded 85% in six studies (Ansah *et al*. 2015; Awor *et al*. 2014, 2015; Maloney *et al*. under review; Mbonye *et al*. 2015; Poyer *et al*. unpublished data; Streat *et al*. Zambia unpublished data). In four studies, ACT provision ranged between 65% and 85% (Allan *et al*. unpublished data; one intervention arm of Maloney *et al*. under review; Streat *et al*. Uganda unpublished data; Onwujekwe *et al*. 2015). The lowest adherence to positive results (30%, 128/421, household surveys) was found in a study in Uganda that also found relatively poor uptake and adherence to negative test results (Cohen *et al*. 2012, 2015).

Eight studies reported on antibiotic use. The proportion of RDT-negative patients receiving antibiotics ranged from 0.3% (6/1854, exit interviews) in a study in Ghana (Ansah *et al*. 2015) to 45% (51/113, household survey) in a study in Uganda (Mbonye *et al*. 2015). Three studies reported antibiotic use exceeding 20% (Cohen *et al*. 2012, 2015; Mbonye *et al*. 2015; Poyer *et al*. unpublished data), with the remaining studies reporting below 20%. Similarly, the proportion of patients with a positive RDT result receiving antibiotics varied from 0% (0/1351, provider records) in Ghana (Ansah *et al*. 2015) to 31% (129/441, household survey) in a study in Uganda (Cohen *et al*. 2012, 2015). Studies that reported relatively high provision of antibiotics to RDT-negative patients also reported high provision to RDT positive patients (Cohen *et al*. 2012, 2015; Mbonye *et al*. 2015; Poyer *et al*. unpublished data).

#### Referrals

Only five studies reported on the proportion of patients who were referred to public hospitals or clinics. In all of these studies, providers were instructed to refer all RDT negative cases. In a study in Ghana (Ansah *et al*. 2015), 62% of the 1088 referred patients interviewed by phone reported attendance at the referral facility. The public health facilities had been made well aware of testing going on in the drug shops and accepted the referred patients. In the remaining four studies, referrals were 10% or less of the cases. Reasons given for low rates of referral were explored explicitly in a qualitative component to the study in Uganda (Mbonye *et al*. 2015), although investigators in other studies reported similar challenges. Providers in the Ugandan study were reluctant to refer except when it was considered medically imperative because of fears that public health workers were unwilling to take patients referred from drug shops or would question the competence of outlet providers, thereby damaging their reputations [34]. Vendors were also concerned that clients might go to another shop rather than to a public facility, and they would lose their clientele. In almost all settings, formal referral systems from private medicine retail outlets to public facilities had not been established. In those studies where providers were encouraged to refer patients with severe illness or if a clear diagnosis could not be made, there was anecdotal evidence of poor or contradictory treatment at the receiving facility.

#### Safety & accuracy in performing RDTs

Nine studies provided data on how RDTs were administered using a check list. In general, most providers were able to accurately perform the RDT, read its results and dispose of the hazardous infectious waste appropriately. In six studies where this outcome was recorded, approximately 85% or more of the providers performed the test safely and correctly (Aung *et al*. 2015; Ansah *et al*. 2015; Awor *et al*. 2014, 2015; Cohen *et al*. 2012, 2015; Maloney *et al*. under review; Mbonye *et al*. 2015). In studies in Nigeria (Streat *et al*. unpublished data) and Uganda (Streat *et al*. unpublished data) only 75% of providers performed and disposed of the RDT appropriately. A study in Liberia (Allan *et al*. unpublished data) found that only 39% (15/38, mystery shoppers) performed all nine of the required steps, with the most common omissions being not disposing of sharps in a safety box and not waiting the appropriate amount of time to read the result. Studies in Tanzania (Maloney *et al*. under review) and Uganda (Cohen *et al*. 2012, 2015) found that providers would often not wear gloves, in addition to the issue of waiting the required number of minutes and adequate disposal of lancets.

#### Price of test to patient

In the seven studies that used a recommended retail price (RRP), actual median retail prices matched the RRP in a study in Tanzania (US$0.67 and US$0.32 in unsubsidized and subsidized arms respectively, Maloney *et al*. under review) and a study in Uganda (at a subsidized price of $1, Streat *et al*. Uganda unpublished results). The actual observed median retail prices exceeded the RRP in the other five studies, ranging from US$0.32 (23% above the RRP) in a study in Liberia (Allan *et al*. unpublished) to US$1.20 (100% above the RRP) in a study in Nigeria (Onwujekwe *et al*. 2015). In a study in Uganda (Cohen *et al*. 2012, 2015) that did not use a RRP, most providers priced RDTs to match local microscopy prices at US$0.40. The remaining studies either provided RDTs for free (Ansah *et al*. 2015; Awor *et al*. 2014, 2015) or for a fixed price of around $0.20 (Mbonye *et al*. 2015; Streat *et al*. Zambia unpublished data).

## Discussion

The introduction of RDTs in PMRs, a primary source of care in many settings, aims to improve case management of febrile illnesses through prompt and appropriate treatment of malaria and a reduction in delays to diagnosis and treatment of other illnesses. This review demonstrates that while RDT introduction can achieve this goal, such outcomes are not guaranteed. Although studies with more intensive interventions generally produced better outcomes, it is unclear whether such efforts could be maintained or scaled up to national level.

The three studies that showed the highest uptake and the highest adherence (Ansah *et al*. 2015; Mbonye *et al*. 2015; Awor *et al*. 2015) included longer trainings (4 or 5 days), close and frequent supervision for an initial period of time (weekly visits by the research team), and low RDT retail prices (US$0.20 or less). These three studies also included the lowest number of outlets compared to the other studies reviewed.

However, there are notable exceptions to these trends. A study in Nigeria (Onwujekwe *et al*. 2015) compared the uptake of RDTs and adherence to national malaria guidelines under different training scenarios and found that longer and more comprehensive training (two days covering diagnosis and treatment versus one day with only a demonstration on how to use RDTs) did not appear to affect uptake or adherence. In contrast, classroom-based trainings on malaria case management in a study in Myanmar (Aung *et al*. 2015), were relatively short (only 0.5 days), but uptake and adherence were better than in some studies with longer trainings. Similarly, in a study in Tanzania (Maloney *et al*. under review), subsidizing the retail price of RDTs by over 50% did not increase uptake compared with an unsubsidized price. Factors that may limit the comparability of outcomes to the intensity of the related interventions include study setting and context (e.g., prior exposure of provider to malaria case management training), the timing of the evaluation (e.g., 3 months vs. 12 months after implementation), the method of data collection (e.g., mystery shopper vs. provider records), the number of outlets included in the studies (e.g., 18 vs. 1502 outlets) or unique events that affected study outcomes (e.g., in a study in Nigeria (Streat *et al*. Nigeria unpublished data), leakage from public sector into the private sector flooded the market, negatively impacting the uptake`of ‘project’ RDTs).

None of the studies deployed interventions that could be scaled-up easily at the national level. For example, a highly effective intervention in Uganda (Mbonye *et al*. 2015) included four day trainings, weekly supervision visits for the first two months and a free, continued supply of RDTs, gloves and sharp boxes. Studies that implemented less intense but perhaps more scalable interventions often produced poorer outcomes. For example, in a study in Uganda (Cohen *et al*. 2012 2015) that showed low RDT uptake and adherence, PMRs were free to choose the price at which the RDTs were sold and free to make treatment recommendations as they wished. Another study, where RDT stock outs were recorded in a study in Tanzania (Maloney *et al*. under review), chose not to control the supply of RDTs; PMRs were simply informed where they could procure RDTs. Schools in an intervention arm of a study in Nigeria (Onwujekwe et al 2015) were supported to organize malaria events to promote uptake and adherence, but only half of the participating schools did so.

Heterogeneity in outcomes following RDT introduction is not unique to the private sector [35–37]. While many public health facilities that increased diagnostic testing for malaria through the use of RDTs also reported reductions in ACT provision, the availability of RDTs alone does not seem sufficient to ensure the appropriate use of ACTs [38, 39]. Public and private providers alike have rationales for providing antimalarials to patients with a negative RDT result. Anxiety over the potential for patients to worsen without being given antimalarials seems paramount [40, 41], just as with antibiotics in other settings [42]. This is accentuated in contexts where antimalarials are expected, or even demanded, by patients or customers [43], and where clients can take their business elsewhere [44]. Overstretched providers may find the time it takes to perform the RDT prohibitive and choose to assist other customers instead of performing the RDT or waiting for its result [45]. In all sectors, behavior change is likely to require sustained efforts.

Experience from these studies showed that requirements for introducing RDTs at scale in PMRs should be viewed as the introduction of a comprehensive service, not just another commodity. However, evidence on how to do so remains limited in many operational aspects. First, evidence is needed on how to integrate malaria testing into case management beyond malaria. Where negative cases are expected to be referred, this may be challenging: clients have chosen the retail sector, providers are keen to make a sale, and public sector workers may be unwelcoming to patients referred from PMRs [34]. Guidelines for managing RDT negative adults and children require specific development, based on levels of expertise, resource availability, and local regulations. Second, evidence is needed on how to train and supervise PMRs, given the size and heterogeneity of the sector as well as rapid staff turnover [13]. It may not be feasible or even desirable to train and supervise all PMRs. Some studies experimented with innovative supervision approaches to prioritize certain shops over others based on sales volume or performance [Poyer *et al*. unpublished data], but little is understood how to find, select or retain these ‘successful’ providers. Some studies in the review [Poyer *et al*. unpublished data; Streat *et al*. Nigeria unpublished data; Streat *et al*. Uganda unpublished data; Maloney *et al*. under review] aimed to provide more sustainable mechanisms (i.e. having professional associations instead of research team members conduct supervision visits, not subsidizing the RDT or controlling the supply chain) that could be scaled and exist beyond the length of the study, but scale up was not tested. New innovative approaches that build on existing structures and programs in the private sector, rather than building parallel infrastructures, require exploration. Third, evidence is needed on how to deal with hazardous waste from testing at scale in these non-clinical settings. In most studies in this review, research teams were responsible for this. One study in Tanzania (Maloney *et al*. under review) that instructed providers to visit public health facilities to drop off waste had mixed success; many providers instead chose to bury or burn their waste. A study in Uganda (Streat *et al*. Uganda unpublished data) contracted a private firm to collect the waste at each of the participating outlets, but poor uptake combined with frequent collection visits caused cost overruns. New innovative approaches to waste disposal require development and evaluation under real world conditions. Finally, additional consideration must also be given to issues beyond malaria control, such as role of PMRs in the wider health system and the legal and regulatory frameworks for *in vitro* diagnostics. A sustained scale up of RDTs in the private retail sector would require recognition from stakeholders, including regulatory bodies, that PMRs are a viable alternative to public sector provision of quality care for uncomplicated malaria.

### Limitations

This review employed a broad search strategy to identify all eligible studies where RDTs were introduced in the private medicine retail sector. We did not include studies that included formal private health facilities such as clinics or hospitals. Since countries are making decisions now about if or how RDTs can be introduced in PMRs, we decided that waiting for more studies to complete the publication process was deemed too much of a delay. While it is possible an eligible but unpublished study could have been missed, this is unlikely given the involvement of extensive contacts identified through the two convened stakeholder meetings in 2013 and 2015 and the large group of authors involved. Some studies did not have data on all the outcomes assessed in the review. The mix of study designs (i.e. differences in intervention and control arm design) and evaluation methods (i.e. mystery shopper or provider records) made formal comparison of point estimates inappropriate. Differences in expectations of RDT positivity and patient demand for diagnosis across studies further limited comparability. The studies included in the review were all small scale trials or pilots with short durations. Most studies evaluated outcomes at a single point in time, which may not be representative of embedded and sustained effects. Finally, studies included in the review did not address the potential regulatory and policy barriers of introducing RDTs to PMRs. All of the studies, except in Myanmar, were provided a waiver to perform RDTs.

## Conclusions

Supporting the introduction of malaria rapid diagnostic testing in private medicine retail outlets has the potential to target antimalarial drugs more effectively. This review shows that a range of private providers in different countries can incorporate RDTs into their practice, although with varying degrees of uptake and influence on case management. This review suggests investment in training and supervision may be beneficial to supporting RDT implementation. However, substantial gaps remain in the evidence for systems that will allow for RDT implementation at scale.

## Supporting information

S1 File PRISMA checklist. (DOC)

S2 File Data sources. (DOCX)
