# Supplementary material for: Introducing malaria rapid diagnostic tests in private medicine retail outlets: A systematic literature review
Source: PLoS One. 2017 Mar 2;12(3):e0173093. doi: 10.1371/journal.pone.0173093 (PMC5333947; doi:10.1371/journal.pone.0173093)
Supplement: S2 File — (DOCX) [file pone.0173093.s002.docx]

**S2. Data Sources**

Eight publications from six studies are available in publicly available data bases. In some instances, we included data from these studies not included in the publications. For these studies, and for data from six unpublished studies that are not (yet) published at the time of this publication, we include contact informations of authors that agreed to provide the underlying data upon request.

Please refer to the following links and contact information to access the underlying data included in the literature review:

1. Ansah EK, Narh-Bana S, Affran-Bonful H, Bart-Plange C, Cundill B, Gyapong M, et al. The impact of providing rapid diagnostic malaria tests on fever management in the private retail sector in Ghana: a cluster randomized trial. BMJ. 2015;350:h1019. doi: 10.1136/bmj.h1019. PubMed PMID: 25739769; PubMed Central PMCID: PMCPMC4353311.

URL: <http://www.bmj.com/content/350/bmj.h1019>

Contact: Evelyn Ansah, [Ansahekdr@yahoo.co.uk](mailto:Ansahekdr@yahoo.co.uk)

1. Awor P, Wamani H, Tylleskar T, Peterson S. Drug seller adherence to clinical protocols with integrated management of malaria, pneumonia and diarrhoea at drug shops in Uganda. Malar J. 2015;14:277. doi: 10.1186/s12936-015-0798-9. PubMed PMID: 26178532; PubMed Central PMCID: PMCPMC4502601.

URL: <https://malariajournal.biomedcentral.com/articles/10.1186/s12936-015-0798-9>

1. Awor P, Wamani H, Tylleskar T, Jagoe G, Peterson S. Increased access to care and appropriateness of treatment at private sector drug shops with integrated management of malaria, pneumonia and diarrhoea: a quasi-experimental study in Uganda. PLoS One. 2014;9(12):e115440. doi: 10.1371/journal.pone.0115440. PubMed PMID: 25541703; PubMed Central PMCID: PMCPMC4277343.

URL: <http://journals.plos.org/plosone/article?id=10.1371/journal.pone.0115440>

1. Aung T, White C, Montagu D, McFarland W, Hlaing T, Khin HS, et al. Improving uptake and use of malaria rapid diagnostic tests in the context of artemisinin drug resistance containment in eastern Myanmar: an evaluation of incentive schemes among informal private healthcare providers. Malar J. 2015;14:105. doi: 10.1186/s12936-015-0621-7. PubMed PMID: 25885581; PubMed Central PMCID: PMCPMC4355503.

URL: <https://malariajournal.biomedcentral.com/articles/10.1186/s12936-015-0621-7>

1. Cohen J, Fink G, Maloney K, Berg K, Jordan M, Svoronos T, et al. Introducing rapid diagnostic tests for malaria to drug shops in Uganda: a cluster-randomized controlled trial. Bull World Health Organ. 2015;93:142–51. doi: 10.2471/BLT.14.142489. PubMed PMID: 25884736; PubMed Central PMCID: PMCPMC4338828.

URL: <http://www.who.int/bulletin/volumes/93/3/14-142489-ab/en/>

Contact: Jessica Cohen, [cohenj@hsph.harvard.edu](mailto:cohenj@hsph.harvard.edu)

1. Cohen J, Fink G, Berg K, Aber F, Jordan M, Maloney K, et al. Feasibility of distributing rapid diagnostic tests for malaria in the retail sector: evidence from an implementation study in Uganda. PLoS One. 2012;7(11):e48296. doi: 10.1371/journal.pone.0048296. PubMed PMID: 23152766; PubMed Central PMCID: PMCPMC3495947.

URL: <http://journals.plos.org/plosone/article?id=10.1371/journal.pone.0048296>

Contact: Jessica Cohen, [cohenj@hsph.harvard.edu](mailto:cohenj@hsph.harvard.edu)

1. Mbonye AK, Magnussen P, Lal S, Hansen KS, Cundill B, Chandler C, et al. A Cluster Randomised Trial Introducing Rapid Diagnostic Tests into Registered Drug Shops in Uganda: Impact on Appropriate Treatment of Malaria. PLoS One. 2015;10(7):e0129545. doi: 10.1371/journal.pone.0129545. PubMed PMID: 26200467; PubMed Central PMCID: PMCPMC4511673.

URL: <http://journals.plos.org/plosone/article?id=10.1371/journal.pone.0129545>

Contact: Sham Lal, [sham.lal@lshtm.ac.uk](mailto:sham.lal@lshtm.ac.uk)

1. Onwujekwe O, Mangham-Jefferies L, Cundill B, Alexander N, Langham J, Ibe O, et al. Effectiveness of Provider and Community Interventions to Improve Treatment of Uncomplicated Malaria in Nigeria: A Cluster Randomized Controlled Trial. PLoS One. 2015;10(8):e0133832. doi: 10.1371/journal.pone.0133832. PubMed PMID: 26309023; PubMed Central PMCID: PMCPMC4550271.

URL: <http://journals.plos.org/plosone/article?id=10.1371/journal.pone.0133832>

Contact: Virginia Wiseman, Virginia.Wiseman@lshtm.ac.uk

1. Allan R, Pontarollo J, Eves K. Establishing malaria case management services in private sector medicine stores serving city slums. Unpublished results. 2015.

Contact: Julie Pontarollo, [julie@mentor-initiative.net](mailto:julie@mentor-initiative.net)

1. Maloney K, Ward A, Krez B, Bryson L, Visser T, LeMenach A, et al. Testing the uptake of low-cost Rapid Diagnostic Tests for malaria in the private sector in Tanzania. Unpublished results. 2015

Contact: Kathleen Maloney, [kmm.maloney@gmail.com](mailto:kmm.maloney@gmail.com)

1. Streat E, Sjoblam M, Yadav P, Freidman J. Increasing access to malaria diagnosis and treatment in Zambia. Unpublished results. 2015.

Contact: Elizabeth Streat, [e.streat@malariaconsortium.org](mailto:e.streat@malariaconsortium.org)

1. Poyer S, Musuva A, Njoki N, Charman N. Increasing access to malaria diagnosis in the private sector on the Kenyan coast. Unpublished results. 2015.

Contact: Stephen Poyer, [spoyer@psi.org](mailto:spoyer@psi.org)

1. Streat E. Increasing access to malaria diagnosis in the private sector in Nigeria. Unpublished results. 2016.

Contact: Elizabeth Streat, [e.streat@malariaconsortium.org](mailto:e.streat@malariaconsortium.org)

1. Streat E. Increasing access to malaria diagnosis in the private sector in Uganda. Unpublished results. 2016.

Contact: Elizabeth Streat, [e.streat@malariaconsortium.org](mailto:e.streat@malariaconsortium.org)
